# Ionic Liquid-Polymer Nanoparticle Hybrid Systems as New Tools to Deliver Poorly Soluble Drugs

**DOI:** 10.3390/nano9081148

**Published:** 2019-08-10

**Authors:** Ana Júlio, Rita Caparica, Sofia A. Costa Lima, Ana Sofia Fernandes, Catarina Rosado, Duarte M. F. Prazeres, Salette Reis, Tânia Santos de Almeida, Pedro Fonte

**Affiliations:** 1 CBIOS—Universidade Lusófona Research Center for Biosciences & Health Technologies, Campo Grande 376, 1749–024 Lisboa, Portugal; 2 Department of Biomedical Sciences, University of Alcalá, Ctra. Madrid-Barcelona Km. 33.600, 28871 Alcalá de Henares, Madrid, Spain; 3 LAQV, REQUIMTE, Department of Chemical Sciences—Applied Chemistry Lab, Faculty of Pharmacy, University of Porto, Rua de Jorge Viterbo Ferreira, 228, 4050-313 Porto, Portugal; 4 iBB-Institute for Bioengineering and Biosciences, Department of Bioengineering, Instituto Superior Técnico, Universidade de Lisboa, 1049-001 Lisboa, Portugal

**Keywords:** ionic liquid, polymer, PLGA, nanoparticle, poorly soluble drug, rutin, drug delivery, hybrid system

## Abstract

The use of functional excipients such as ionic liquids (ILs) and the encapsulation of drugs into nanocarriers are useful strategies to overcome poor drug solubility. The aim of this work was to evaluate the potential of IL-polymer nanoparticle hybrid systems as tools to deliver poorly soluble drugs. These systems were obtained using a methodology previously developed by our group and improved herein to produce IL-polymer nanoparticle hybrid systems. Two different choline-based ILs and poly (lactic-co-glycolic acid) (PLGA) 50:50 or PLGA 75:25 were used to load rutin into the delivery system. The resulting rutin-loaded IL-polymer nanoparticle hybrid systems presented a diameter of 250–300 nm, with a low polydispersity index and a zeta potential of about −40 mV. The drug association efficiency ranged from 51% to 76%, which represents a good achievement considering the poor solubility of rutin. No significant particle aggregation was obtained upon freeze-drying. The presence of the IL in the nanosystem does not affect its sustained release properties, achieving about 85% of rutin released after 72 h. The cytotoxicity studies showed that the delivery system was not toxic to HaCat cells. Our findings may open a new paradigm on the therapy improvement of diseases treated with poorly soluble drugs.

## 1. Introduction

Ionic liquids (ILs) are organic salts [1,2], which combine an organic cation and an organic/inorganic anion [1,3], that are liquid below 100 °C [4,5]. These salts have been classified into four categories, accordingly to the cation present in these ILs, namely as, dialkylimidazolium, *N*-alkyl-pyridinium, phosphonium, or alkylammonium cation [6,7,8,9]. Amongst these classes, imidazolium-based ILs are the most studied, due to their high stability, low viscosity [6,7,8] and it is relatively easy synthesis [6,7,8]. However, in the drug delivery field, they have exhibited some limitations, since they have shown high toxicity [8,10,11]. In contrast, the quaternary ammonium-based ILs, such as the choline-based ILs, have been described as the less toxic [4,8,11,12], so they have been considered in the literature as “green” ILs [7,8,12,13]. This fact puts them at the forefront as more suitable for applications in the pharmaceutical field [8,11,12,13].

In general, ILs have several distinct and valuable properties, such as high thermal and chemical stability, low vapour pressure, non-volatility, the possibility of being recycled and the ability to be solubilized in several solvents [7,8,12,14]. Due to these properties and to the ability to be modified according to a desired physicochemical property, they may be used for different purposes [1,6,8]. For instance, ILs have been applied in different fields, including in several organic reactions [15,16,17,18,19,20], in extractions and separation reactions [15,21,22,23,24], in electrolysis and electrochemistry [25,26,27,28] and in nanotechnology [9,28,29,30,31]. Another emergent application of ILs is as functional excipients, due to their ability to increase drug solubility and/or permeation and as drug stabilizers [1,2,3,5,8,12,32,33,34]. Some studies have established the value of ILs as solubility enhancers for topical formulations, like gels and emulsions [8,12,33,35,36]. However, only a few studies have considered the incorporation and functionality of these ILs at non-toxic concentrations [8,12,34]. Hence, evaluating the maximum concentration of these ILs that does not impact cell viability, and prove their functionality, at these concentrations is crucial to prove safety alongside with the ability to increase the efficiency of the delivery systems.

The encapsulation of drugs into nanoparticles have been also a useful strategy to protect the drug while allowing a controlled and/or targeted drug delivery to a specific tissue, improving its bioavailability and decreasing adverse side effects [37,38,39,40,41]. In addition, nanoencapsulation also enables the incorporation of hydrophobic and hydrophilic drugs while being well tolerated through different routes of drug delivery [42,43]. Several types of polymer nanoparticles have been used due to its good biocompatible and biodegradable properties [42,44,45,46]. Such carriers have shown the ability to increase the absorption, bioavailability, solubility, and stability of drugs [9,47,48,49]. Furthermore, they may also be tailored according to the required application [9,47,48,49].

The poly(lactic-co-glycolic acid) (PLGA) is a polymer approved by the Food and Drug Administration (US FDA) and by the European Medicine Agency (EMA) for application in nanomedicine [44,45,46,50]. It has high biodegradability and biocompatibility and shows a good controlled release profile over time [44,45,46,47,48,49,50,51,52,53]. The common PLGA ratios used in drug delivery are 50% lactic acid and 50% glycolic acid (PLGA 50:50) and 75% lactic acid to 25% glycolic acid (PLGA 75:25) [44,46]. These ratios are commonly used due to their easier degradation in the human body since the combination of lactic acid and glycolic acid is quickly hydrolyzed to the monomers [44]. The PLGA nanoparticles are used with several applications, such as vaccination, cancer, and other diseases [44].

Therefore, the combination of polymeric nanoparticles with ILs may be a valuable strategy by taking advantage of the synergistic effects of both materials, leading to delivery systems with higher physicochemical and colloidal stability [9,54,55,56], and increased the drug loading. More importantly, this combination may improve the therapeutic effect of poorly soluble drugs while decreasing possible adverse side effects. A good example of a poorly soluble drug is rutin, with a solubility in water of 0.2 mg/mL [12,19,35,37]. Rutin is a polyphenolic bioflavonoid extracted from fruits and plants [57,58]. Its antioxidant, antidiabetic activity, antihypertensive, and antilipidemic activity are widely reported in the literature [58,59]. Additionally, in vitro studies also demonstrated that rutin may have an anticancer effect (as leukaemia preventive agent and as inhibitor of human adenocarcinoma in HT-29 and Caco-2 cells), an anti-inflammatory effect (against cyclooxygenase and lipoxygenase) and a neuroprotector effect [12,57,58,59,60]. However, its applicability is limited since its bioactivity is impaired by the poor solubility, stability, and permeability [12,58,59,60].

Previously, we developed a new IL-polymer nanoparticle hybrid system to load poorly soluble drugs [39]. Herein, the aim of this work was to take this approach further by improving this new nanocarrier and deeper evaluate its performance in terms of its physicochemical features, drug delivery profile, and cytotoxicity characteristics. Such drug delivery system may be a valuable tool to deliver poorly soluble drugs by combining the advantages of both ionic liquids and nanocarriers.

## 2. Materials and Methods

### 2.1. Materials

Concerning the synthesis of ILs, choline hydroxide in methanol [Cho][OH]/MeOH 45% was purchased from Sigma-Aldrich (St. Louis, MO, USA), acetonitrile was from VWR (Fontenay-sous-Bois, France) and methanol was from Sigma-Aldrich Chemie Gmbh (Munich, Germany). The amino acids used L-phenylalanine was obtained from PanReac AppliChem^®^ ITW Reagents (Barcelona, Spain) and L-glutamine from Sigma-Aldrich (St. Louis, MO, USA). The rotatory evaporator used was an IKA RV06-ML from IKA^®^-Werke GmbH and Co. (Staufen, Germany) and the centrifuge was a Hermle Z 32 HK from Hermle LaborTechnik (Wehingen, Germany).

Regarding the production of the nanoparticles, two different ratios of PLGA were used, 50:50 (Purasorb^®^ PDLG 5002A) and 75:25 (Purasorb^®^ PDLG 7502A), that were kindly supplied by Corbion Purac (Amsterdam, The Netherlands). Dichloromethane and polyvinyl alcohol (PVA) from Sigma-Aldrich (St. Louis, MO, USA) were also used. Rutin was purchased from Fagron (São Paulo, Brazil) and trehalose was from PanReac AppliChem^®^ ITW Reagents (Barcelona, Spain). The bidistilled water was prepared in-house.

For cytotoxicity studies, trypsin, penicillin-streptomycin solution, fetal bovine serum, dimethyl sulphoxide (DMSO), and thiazolyl blue tetrazolium bromide (MTT) were purchased from Sigma-Aldrich (St. Louis, MO, USA) and Dulbecco’s modified Eagle’s medium (DMEM) was provided by Biowest (Nuaillé, France). Finally, in the permeation study, it was also used absolute ethanol from Sigma-Aldrich (St. Louis, MO, USA).

### 2.2. Synthesis of ILs

Two choline-based ILs, (2-hydroxyethyl)-trimethylammonium-L-phenylalaninate [Cho][Phe] and (2-hydroxyethyl)-trimethylammonium-L-glutaminate [Cho][Glu] were synthesized as previously described in the literature [8]. Briefly, an aqueous solution of 57.79 mmol of the amino acid was added to 15.6 mL of [Cho][OH]/MeOH 45%, previously evaporated at 50 °C under vacuum. The obtained mixture was cooled in an ice bath under stirring (17 h) and subsequently the water was evaporated at 60 °C under vacuum. After that, the unreacted amino acid was precipitated using a mixture of acetonitrile:methanol (9:1), under vigorous stirring. The obtained solid was removed by centrifugation at 10,080× *g* for 20 min, followed by a gravimetric filtration. The solvents were then evaporated under vacuum at 60 °C. The synthesized IL was stored under moisture-free conditions until use. The ILs were characterized by ^1^H NMR and ^13^C NMR at 400 MHz using D_2_O as a solvent, in a Brucker Avance 400 from Bruker Corporation (Billerica, MA, USA).

### 2.3. Production of the IL-Polymer Nanoparticle Hybrid Systems

The IL-polymer nanoparticle hybrid system was produced using a water in oil in water (W/O/W) double emulsion technique described by Júlio et al. [39] with slight modifications. Briefly, 200 µL of an aqueous solution of rutin in 0.2% (*v*/*v*) of each IL [8], [Cho][Phe] or [Cho][Glu], was prepared, dissolving the drug proximal to its saturation point (1.15 mg/mL for [Cho][Phe] and 0.68 mg/mL for [Cho][Glu] [12]). Then, dichloromethane was added to 200 mg of PLGA (50:50 or 75:25) and the water:IL solution containing rutin was blended with the PLGA mixture. These samples were then sonicated for 30 s at 70% of amplitude using a Q125 Sonicator from QSonica Sonicators (Newtown, CT, USA). This first emulsion was immediately poured into 25 mL of PVA 2% (*w*/*v*) [39] and directly sonicated under the same previous conditions. Finally, the formulation was placed under magnetic stirring until organic solvent removal. All formulations were produced in triplicate.

### 2.4. Particle Size, Polydispersity Index and Zeta Potential

The particle size and polydispersity index (PdI) were analyzed by dynamic light scattering and its zeta potential was evaluated by the electrophoretic mobility technique using a Delsa™ Nano C from Beckman Coulter, Inc. (Brea, CA, USA). All samples were run in triplicate at room temperature (23 ± 2 °C), after proper dilution with bidistilled water.

### 2.5. Association Efficiency (AE) of Rutin

The AE is a parameter that quantifies the amount of drug associated with nanoparticle systems. The formulations were centrifuged at 16,350× *g* for 15 min at 4 °C, and the supernatant was collected. The rutin present in the supernatant was quantified by UV spectroscopy using a UV–VIS Spectrophotometer Evolution^®^ 300 from Thermo Scientific (Hertfordshire, England) at its maximum absorption wavelength (354 nm).

The AE of rutin was calculated using the Equation (1):

(1) $$\mathrm{AE}\;=\;\frac{\mathrm{Total}\;\mathrm{amount}\;\mathrm{of}\;\mathrm{rutin}-\mathrm{Free}\;\mathrm{amount}\;\mathrm{of}\;\mathrm{rutin}\;\mathrm{in}\;\mathrm{the}\;\mathrm{supernatant}}{\mathrm{Total}\;\mathrm{amount}\;\mathrm{of}\;\mathrm{rutin}}\times 100\;$$

### 2.6. Freeze-Drying of the IL-Polymer Nanoparticle Hybrid Systems

The formulations were centrifuged at 12,600× *g* for 15 min at 4 °C in Hermle Z 32 HK centrifuge to remove the supernatant containing PVA at 2% (*w*/*v*), and the particles were redispersed in a solution of trehalose at 3% (*w*/*v*). Samples without lyoprotectant were also prepared.

The formulations were frozen overnight at −80 °C, and freeze-dried in a Labconco FreeZone 25^®^ (Kansas City, MO, USA) at a surface condenser temperature of −50 °C and 400 mTorr for 48 h.

### 2.7. Reconstitution of the Lyophilizates and Freeze-Drying Ratio

The freeze-dried samples were reconstituted by adding bidistilled water in the inside wall of the glass flask and maintained for 5 min. to ensure the cake wetting, and slowly shaken until complete homogenization. Then, the particle size was characterized using the methodology described above and the freeze-drying ratio was calculated using the Equation (2). The freeze-drying ratio is a parameter that allows to understand the maintenance of the physicochemical features of the nanoparticles upon freeze-drying:

(2) $$\mathrm{Freeze}-\mathrm{drying}\;\mathrm{ratio}\;=\;\frac{\mathrm{Mean}\;\mathrm{particle}\;\mathrm{size}\;\mathrm{after}\;\mathrm{freeze}-\mathrm{drying}}{\mathrm{Mean}\;\mathrm{particle}\;\mathrm{size}\;\mathrm{before}\;\mathrm{freeze}-\mathrm{drying}}$$

### 2.8. Fourier Transform Infrared Spectroscopy (FTIR)

The IL-polymer nanoparticle hybrid systems obtained after freeze-drying was evaluated by FTIR in a PerkinElmer^®^ Spectrum 400 (Waltham, MA, USA) equipped with an attenuated total reflectance (ATR) device. The spectra were obtained collecting 100 scans of each sample, between 4000 and 600 cm^−1^, with a resolution of 4 cm^−1^. The FTIR analysis was also performed for rutin and other control samples.

### 2.9. Differential Scanning Calorimetry (DSC)

The thermograms of freeze-dried formulations were obtained using a Differential Scanning Calorimeter DSC 200 F3 Maia Netzsch^®^ (Selb, Germany). Samples were weighed (1 mg) and placed into Netzsch^®^ aluminum pans (Selb, Germany), which were hermetically sealed, and the thermal analysis was performed in a temperature range between 20 °C and 100 °C, with a rate of 10 °C per minute.

### 2.10. Scanning Electron Microscopy (SEM)

The SEM analysis of the resuspended freeze-dried samples was performed on a JSM-7001F from JEOL (Tokyo, Japan) after they were put onto metal stubs and vacuum-coated with a layer of gold/palladium during 20 s with a current of 25 mA. The samples were previously resuspended in bidistilled water and washed at 12,600× *g* for 15 min at 4 °C in a Hermle Z 32 HK centrifuge from Hermle LaborTechnik (Wehingen, Germany) to remove the surfactant, that is dissolved in the bidistilled water.

### 2.11. In Vitro Release Study

For in vitro release study of rutin, the nanoparticle suspension was centrifuged at 12,600× *g* for 20 min at 4 °C, and the obtained pellet was resuspended in 10.0 mL of pH 7.4 phosphate buffer saline (PBS) solution. These solutions were incubated at 37 °C with stirring at 100 rpm in a Heidolph^®^ 1000 incubator with a motor Heidolph^®^ Unimax 1010 (Schwabach, Germany). Aliquots of each sample were taken at predetermined time intervals (30 min, 1, 2, 4, 6, 8, 12, 24, 48, and 72 h) and replaced with the same volume of PBS. After samples centrifugation at 12,600× *g* for 15 min in a Hermle Z 32 HK centrifuge, rutin in the samples was quantified in a UV–VIS Spectrophotometer Evolution^®^ 300 from Thermo Scientific (Hertfordshire, England) at a fixed wavelength of 354 nm.

### 2.12. Permeation Study

The permeation studies (*n* = 5) were performed on vertical diffusion glass cells (Franz cells) with a receiver volume of approximately 4 mL and a diffusion area of 0.95 cm^2^, using a polydimethylsiloxane (PDMS) membrane. Thus, 500 μL of the nanoparticle suspension was placed in the donor compartment, which was then occluded with microscope coverslips. The receptor compartment was immersed in a thermostatic bath at 37 °C and was filled with a mixture of PBS pH 7.4:ethanol (80:20).

At predetermined time intervals (3, 6, 9, 12, and 24 h), the medium in the receptor compartment was collected and replaced with a previously thermostated mixture of PBS pH 7.4:ethanol (80:20). After the collection, rutin was quantified using a UV–VIS Spectrophotometer Evolution^®^ 300 from Thermo Scientific (Hertfordshire, England) at a fixed wavelength of 354 nm.

### 2.13. MTT Cytotoxicity Studies

HaCat human keratinocytes were cultured in DMEM supplemented with 10% fetal bovine serum and 1% penicillin-streptomycin. The cells were maintained at 37 °C, under a humidified air atmosphere containing 5% of CO_2_ in air and seeded at a density of 5 × 10^3^ per well in 200 µL culture medium in 96-well plates and incubated for 24 h. The cells were then exposed to the nanoparticle formulations for a 24 h period. The MTT reduction assay was then carried out, according to a previously described protocol [61,62,63]. The absorbance values for cultures incubated only with vehicle (PBS 5% *v*/*v*) corresponds to 100% cell viability. DMSO (5% *v*/*v*) was used as positive control and decreased cell viability to 2.97 ± 1.32%. For this assay, two independent experiments were performed, and at least four replicate cultures were used in each experiment.

### 2.14. Statistical Analysis

The obtained results were evaluated by one-way analysis of variance, ANOVA, followed by Tukey’s multiple comparison tests. The values are expressed as mean ± standard deviation (SD). The differences between samples were significant at *p* < 0.05 level. Results were treated using a GraphPad Prism 5^®^ from GraphPad Software (San Diego, CA, USA).

## 3. Results and Discussion

In this work, we produced an IL-polymer nanoparticle hybrid system following an adapted W/O/W double emulsion technique [39] to load rutin. This system was composed of a choline-amino acid IL, [Cho][Phe] or [Cho][Glu], and PLGA 50:50 or PLGA 75:25 as polymers following a production method developed by our group [39] and improved herein.

It was our aim to demonstrate the ability of ILs to be placed within polymer nanoparticle matrices, achieving robust and stable hybrid delivery systems for multifunctional applications.

The developed formulations result from the combination of the two ratios of PLGA (50:50 or 75:25) with each of the choline-based ILs. Hence, from now on, the formulations combining PLGA 50:50 with [Cho][Phe] or [Cho][Glu], will be referred as PLGA 50:50/[Cho][Phe] and PLGA 50:50/[Cho][Glu], respectively. Furthermore, the formulations combining the PLGA ratio 75:25 with each IL will be assigned as PLGA 75:25/[Cho][Phe] and PLGA 75:25/[Cho][Glu]. Additionally, when these formulations contain rutin the denotation, + Rutin, will be added to account for the presence of the drug.

The amino acid-based ILs were chosen since previous studies have shown that they are able to enhance drug solubility at concentrations where cell viability is maintained—0.2% (*v*/*v*) [8]. Hence, [Cho][Phe] and [Cho][Glu] were synthesized, according to the literature [8], their structure was confirmed by ^1^H-NMR and ^13^C-NMR using D_2_O as a solvent, and the results were found to be in agreement with the literature [4,12]. After the synthesis of the ILs and preparation of the IL-polymer nanoparticle hybrid systems, the obtained delivery systems were evaluated.

### 3.1. Particle Size, Polydispersity Index, and Zeta Potential

**Figure 1:**
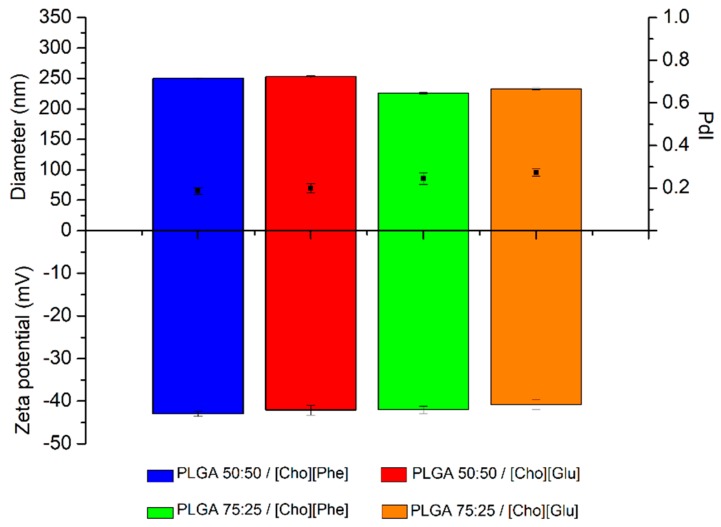
Diameter (nm) (top bars), PdI (black squares), and zeta potential (mV) (bottom bars) of IL-polymer nanoparticle hybrid systems (*n* = 3, mean ± SD).

First, the IL-polymer nanoparticle hybrid system without rutin was produced to understand the interaction of the choline-based ILs with the polymers and evaluate the possibility to obtain nanoparticles. Thus, the diameter, PdI and zeta potential of these formulations were evaluated. The diameter ranged between 200–250 nm, the PdI was around 0.3, whereas the zeta potential was about −40 mV (Figure 1), which are appropriate properties for the administration of drugs by different administration routes. The robustness of the production method to obtain nanoparticles with similar characteristics was also confirmed. It was also demonstrated, for the first time, that the choline-based ILs placed within unloaded polymer nanocarriers allow to obtain stable and robust nanoparticles, since the results of the IL-polymer nanoparticle hybrid systems were similar those previously described for polymer nanoparticles without ILs [51,52,64].

**Figure 2:**
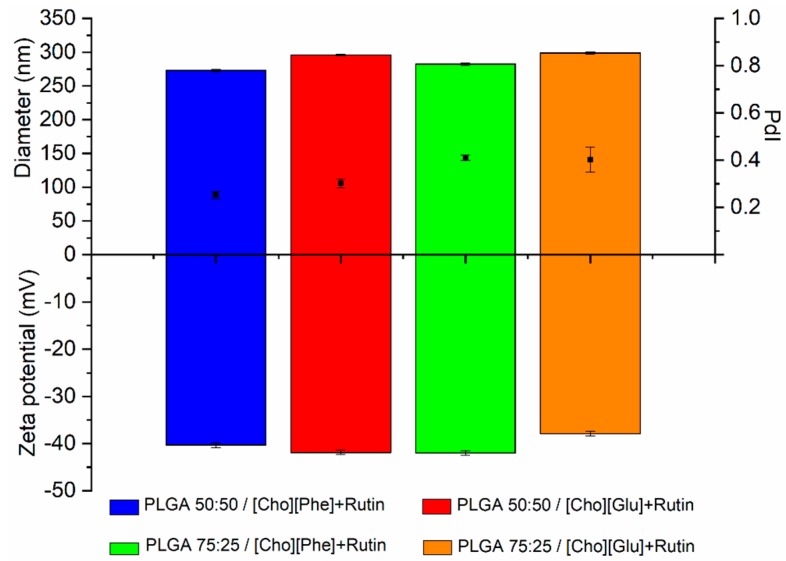
Diameter (nm) (top bars), PdI (black squares), and zeta potential (mV) (bottom bars) of rutin-loaded IL-polymer nanoparticle hybrid systems (*n* = 3, mean ± SD).

Then, the rutin-loaded IL-polymer nanoparticle hybrid systems were prepared in the presence of ILs since the low aqueous solubility of the drug (around 0.2 mg/mL [12,57,60]) hampers its encapsulation in the absence of the ILs. Hence, the ILs were used, separately, to achieve maximum solubility for rutin in 0.2% (*v*/*v*) of [Cho][Glu] or [Cho][Phe], which corresponds to 0.68 mg/mL and 1.15 mg/mL, respectively [12]. These rutin-loaded formulations showed a particle size ranging from 250 to 300 nm with a PdI between 0.3 and 0.4 and a zeta potential of about −40 mV (Figure 2). These results show the robustness of the developed hybrid systems since they are similar to those obtained without the drug (Figure 1). Additionally, it was observed a slight increase in diameter and in the PdI, that indicates the presence of rutin in the nanocarrier. Which is also corroborated by the FTIR and DSC results.

The IL-polymer nanoparticle hybrid system, with and without rutin, demonstrated good colloidal stability, and high negative charge (Figure 1 and Figure 2). Additionally, no significant differences were detected between the analyzed parameters, thus confirming that the encapsulation of rutin does not change the physicochemical properties of the IL-polymer nanoparticle hybrid systems (compare data in Figure 1 and Figure 2).

To extend the shelf life of the delivery system [51,52], the IL-polymer nanoparticle hybrid system was freeze-dried using trehalose 3% (*w*/*v*) as lyoprotectant. To evaluate the impact of this process in the nanocarrier system, the freeze-drying ratio was calculated according to Equation (2). The results showed that the diameter of the IL-polymer nanoparticle hybrid system did not significantly change in the presence of trehalose, since the ratio was close to 1.00 in all samples (Table 1). This may indicate that the incorporation of the IL in the nanocarriers does not interfere with the lyoprotectant effect of trehalose [51].

Despite the formulations without trehalose had a freeze-drying ratio higher than 1, no particle aggregation was observed showing once again the stability of the system. This result is also indicative that the presence of the IL in the nanoparticle matrix contributed to decrease the stress effect of the freeze-drying process on the nanoparticles, since all formulations had a ratio around 1.13 and 1.50, which is much lower to what happens in the absence of the ILs [47,65]. This can be explained by the possibility of ILs act as water substitutes [66], which is a protective mechanism of lyoprotectants.

Moreover, since all samples presented a freeze-drying ratio closed to 1.00, this shows that the hybrid nanosystems, with and without the lyoprotectant did not suffer from stress [65], since they kept their particle size after freeze-drying, without any noticeable signs of particle aggregation or damage.

### 3.2. Association Efficiency (AE) of Rutin

Comparatively to our previous work, an improvement of about 20% in the AE of rutin in the IL-polymer nanoparticle hybrid systems was obtained for both ILs (Table 2). These results represent a considerable achievement in the incorporation of a poorly soluble drug into the nanoparticles without compromising the particle size. This shows that the presence of 0.2% (*v*/*v*) of a choline-based IL in the nanoparticle is determinant to load rutin in the hybrid system with a high AE. Additionally, the results also showed that there are no statistically significant differences in the AE for the two ratios of PLGA. More importantly, the formulations with [Cho][Phe] demonstrated significantly higher AE values than formulations containing [Cho][Glu] (*p* < 0.05), which indicates it might be a better IL to allow the incorporation of high amounts of poorly soluble drugs. These results may be explained by the higher aqueous solubility of rutin in the presence of [Cho][Phe], compared to [Cho][Glu], which explains the higher AE of the drug in the presence of the phenylalaninate IL.

### 3.3. Fourier Transform Infrared Spectroscopy (FTIR) Analysis

**Figure 3:**
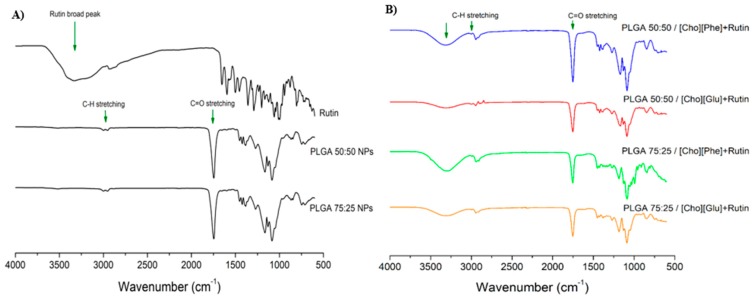
FTIR spectra of controls (Rutin, PLGA 50:50 and PLGA 75:25) (**A**) and rutin-loaded IL-PLGA nanoparticle hybrid systems (**B**), all obtained after freeze-drying.

FTIR spectra were collected for rutin, and both PLGA ratios (50:50 and 75:25) to serve as controls (Figure 3A) and for the rutin-loaded IL-polymer nanoparticle hybrid systems (Figure 3B). All analyses were performed after samples freeze-drying. The PLGA spectrum showed the presence of the characteristic peak between 1750–1760 cm^−1^, which corresponds to the C=O stretching (Figure 3) [67,68,69] and the peak at 3000 cm^−1^, corresponding to the C–H stretching (Figure 3) [67,68,69]. The comparison between the spectra of PLGA (Figure 3A) and of the rutin-loaded IL-polymer nanoparticle hybrid systems (Figure 3B) reveals an increase in the intensity of the C=O and C–H bands, which may be caused by the superimposition of the rutin peaks at the same wavenumber [70,71,72]. Furthermore, the IL-polymer nanoparticle hybrid systems also display a band between 3250 and 3400 cm^−1^ (Figure 3B), which is similar to the characteristic broad peak of rutin [70,71,72], also observed at the individual spectrum for this drug (Figure 3A). This observation points to the existence of an interaction between the drug and the IL-polymer nanoparticle hybrid systems, which may show that rutin was efficiently encapsulated. It was also observed that this broad peak was increased in the formulations with higher AE (Table 2), namely in PLGA 50:50/[Cho][Phe] and PLGA 75:25/[Cho][Phe].

### 3.4. Differential Scanning Calorimetry (DSC) Analysis

**Figure 4:**
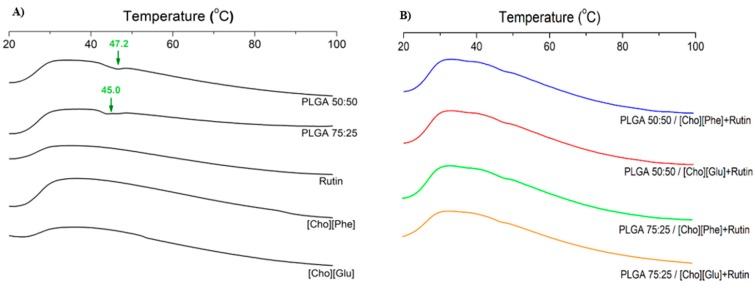
DSC thermogram of controls (PLGA 50:50, PLGA 75:25, rutin, [Cho][Phe] and [Cho][Glu]) (**A**), rutin-loaded IL-PLGA nanoparticle hybrid systems (**B**), all obtained after freeze-drying.

Polymer-drug and/or polymer-IL interactions were evaluated by DSC analysis. DSC analysis was performed both for rutin-loaded IL-polymer nanoparticle hybrid systems and for the controls—PLGA 50:50, PLGA 75:25, [Cho][Phe], [Cho][Glu] and rutin. The thermograms showed that the melting profiles of all samples are similar (Figure 4) and that the interactions between the compounds do not interfere with the behavior of each compound under increasing temperature (Figure 4B). Moreover, the characteristic endothermic peak of PLGA at 47.2 °C for 50:50 ratio and 45.0 °C for 75:25 ratio [34,69,71,73], appears on the control samples (Figure 4A). Additionally, the incorporation of the ILs in the polymer nanoparticles seems to stabilize the nanocarriers, since the endothermic peak of PLGA is less pronounced or even insubstantial in the IL-polymer nanoparticle hybrid systems (Figure 4B) than in the PLGA controls (Figure 4A). This phenomenon is also verified in the unloaded IL-polymer nanoparticle hybrid systems (data not shown). Yet, rutin, at the analyzed temperature, was not altered in the presence of the developed delivery system when compared with the thermogram of the rutin control (Figure 4). Finally, the DSC profiles of both ILs, in the hybrid nanocarriers, were also similar (Figure 4B) to the respective controls (Figure 4A).

### 3.5. Scanning Electron Microscopy (SEM) Analysis

The unloaded and rutin-loaded IL-polymer nanoparticle hybrid systems were analyzed by SEM, after freeze-drying without lyoprotectant. Analysis of the PLGA nanoparticles produced by the same method, but in the absence of ILs, was also performed to be used as control.

**Figure 5:**
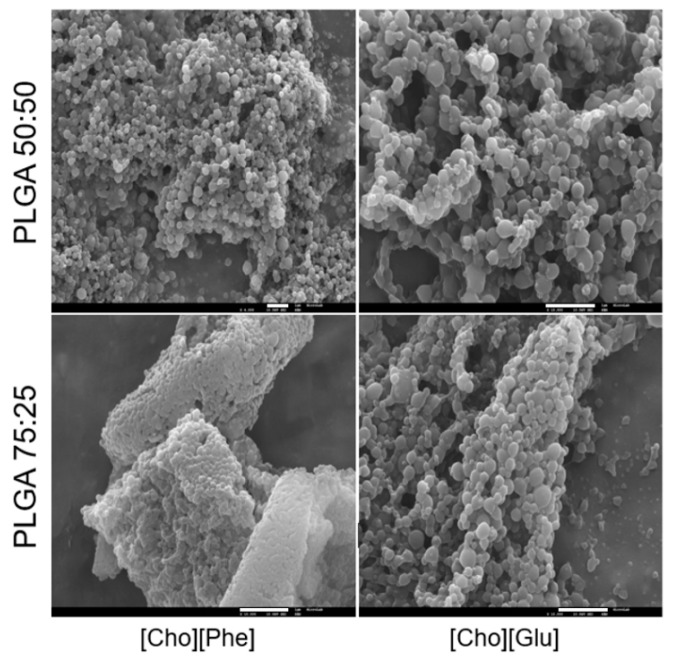
SEM microphotographs of rutin-loaded IL-polymer nanoparticle hybrid systems after freeze-drying at a magnification of 4000× (PLGA 50:50/[Cho][Phe]) and 10,000× (remaining images). The scale bar of the microphotographs at the bottom right of the images corresponds to 2 µm.

The SEM analysis showed that the incorporation of the IL on the inner phase of the nanosystem does not interfere with the nanoparticle morphology since the PLGA nanoparticles and the IL-polymer nanoparticle hybrid systems display a similar spherical morphology with a smooth surface (Figure 5), which is in agreement with the literature for this type of nanoparticles without IL [39,51,52]. Furthermore, the similarity in particle size observed in these analyses (Figure 5) was also concordant with the physicochemical properties previously determined herein (Figure 1 and Figure 2).

### 3.6. In Vitro Rutin Release Study

**Figure 6:**
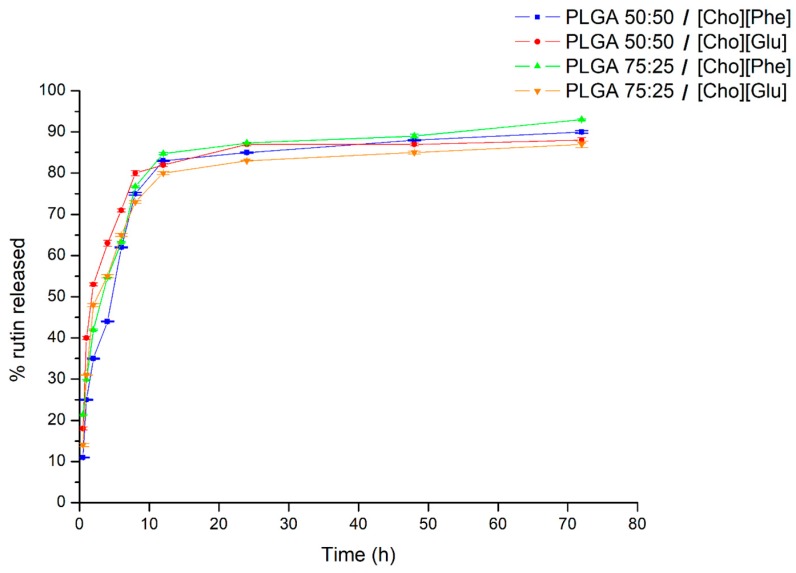
Release profile of the rutin-loaded IL-PLGA nanoparticle hybrid systems during 72 h in phosphate buffer saline at pH 7.4. Data represented as mean ± SD (*n* = 3).

The release of rutin from the IL-polymer nanoparticle hybrid systems was studied using freshly prepared formulations. All formulations demonstrated a controlled release profile of rutin (Figure 6), which is a typical PLGA pattern [47,52].

There is an initial burst in the first 2 h of the study (Figure 6), which is likely to be caused by rutin adsorbed to the surface of the nanoparticles [51,74,75]. After this initial burst, the hybrid systems presented a sustained drug release over time (Figure 6), reaching values between 85% to 95%, after 72 h (Figure 6). Additionally, there is not significant differences in the release profile between both PLGA ratios neither between choline-based ILs. These results demonstrate the ability of the IL-polymer nanoparticle hybrid systems to deliver rutin in a sustained manner up to 72 h. More importantly, it was demonstrated that the presence of ionic liquids in the nanoparticles does not hamper the drug release by keeping the characteristic sustained release.

### 3.7. Permeation Study

The permeation study was performed in vitro with a PDMS membrane. Taking into account the low water solubility of rutin and according to the method described in the literature [76], the receptor fluid was a mixture of phosphate buffer pH 7.4 and ethanol (80:20), to guarantee that the assay was done in sink conditions [76].

The permeation assay was performed using all formulations and an aqueous solution of rutin, containing each IL, at the same concentration as in the nanocarrier formulation was used as control. After 24 h, the results showed that free and encapsulated rutin presented a low skin permeation, with the permeation flux between 0.48 and 0.55 µg/cm^2^/h (Table 3), which may be explained by the high molecular weight and the high hydrophobicity of rutin [57].

Although no significant differences between the sample and the control were found, the higher lipophilicity of rutin [57] may contribute to the permanence of the drug on the PDMS surface or decrease in its release to the medium receptor, since PDMS membrane has also higher lipophilic properties [77,78]. These results may indicate that these systems can be used for topical administration.

### 3.8. MTT Cytotoxicity Assay

Given that rutin may be used topically [12,57,58], a study was performed with HaCat human keratinocytes to check the impact of the IL-polymer nanoparticle hybrid systems on the cell viability. The MTT reduction assay, which is a colorimetric assay that quantifies the mitochondrial function, measured the effect of the system [62].

To understand if the presence of the IL in the nanocarrier interferes with the viability of HaCat cells, a comparative analysis was performed with unloaded PLGA nanoparticles in the absence of the IL and unloaded IL-polymer nanoparticle hybrid systems. Furthermore, we also aimed to investigate whether drug encapsulation in the IL-polymer nanoparticle hybrid systems could cause changes in the interaction with HaCat. Therefore, a rutin-loaded IL-polymer nanoparticle hybrid system was also evaluated alongside with an aqueous solution of rutin containing ILs. Additionally, to go even further, we also tested the samples obtained at the end of the release study, which we refer to hereafter as leachable.

It is also important to mention that, previous studies in our group already showed that both choline-based ILs used maintained the cell viability in these cells at the same tested conditions until 0.2% (*v*/*v*) [8].

**Figure 7:**
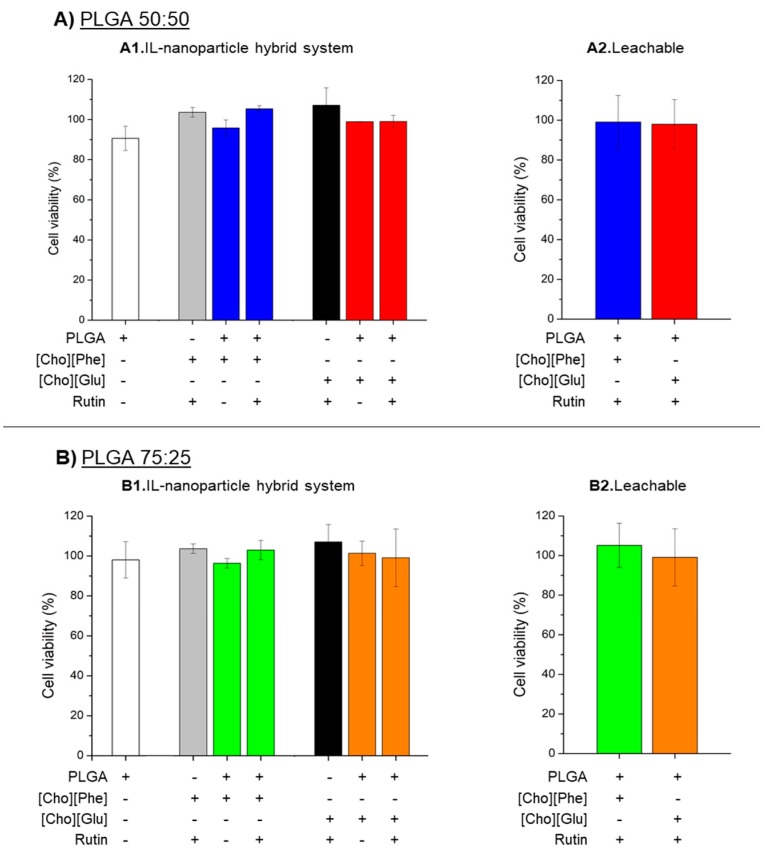
Cell viability of HaCat cells exposed to unloaded PLGA nanoparticles, rutin-loaded choline-based IL-polymer nanoparticle hybrid systems, an aqueous solution of rutin with 0.2% (*v*/*v*) of choline-based IL (**1**) and leachable (**2**). The presented ratios PLGA were 50:50 (**A**) and 75:25 (**B**) and concentration of rutin is 0.29 µM and 0.69 µM for samples containing [Cho][Glu] and [Cho][Phe], respectively. In all samples, the cell viability after 24 h was evaluated by MTT reduction assay. Values represent mean ± SD (*n* = 2) and are expressed as a percentage of the non-treated control cells.

Regarding the obtained results, they showed that, under our experimental conditions, there was no significant difference between the unloaded PLGA nanoparticles and the PLGA nanoparticles combined with the ILs (Figure 7(A1,B1)). This indicates that the presence of the ILs as the solubility promotor in the inner phase of the nanoparticles does not decrease cell viability. Additionally, viability remained unaffected after cell exposure to IL-polymer nanoparticle hybrid systems and to an aqueous solution of rutin with 0.2% (*v*/*v*) of ILs. The experiments performed with the leachable further showed that the components leached from a 72 h contact with rutin-loaded IL-polymer nanoparticles do not cause damage to cells (Figure 7(A2,B2)), suggesting the biocompatibility of the new developed hybrid systems.

## 4. Conclusions

The development of drug delivery systems faces several challenges, such as the low aqueous solubility and permeability of some drugs. Then, the combination of ILs and the nanoencapsulation may be a strategy to overcome these drawbacks. Thus, in this work, two different amino acid-based ILs were used, [Cho][Phe] and [Cho][Glu], as solubility enhancers of a poorly soluble drug, rutin, for the development of IL-polymer nanoparticle hybrid systems.

The physicochemical characterization of the hybrid nanosystems proved that this combination contributes to a particle size between 250 and 300 nm, with good polydispersity and high colloidal stability. Besides that, it was possible to obtain an association efficiency higher than 50% for both ILs and up to about 76% in the presence of [Cho][Phe], which is a significant achievement considering the low aqueous solubility of rutin. This significant improvement was attained while maintaining the stability and particle size of the developed hybrid systems. Furthermore, the nanosystems did not shown any significant particle aggregation upon freeze-drying. Such results demonstrated the robustness of the delivery system

It was observed that the developed nanocarriers had a sustained release up to 72 h, demonstrating that the presence of the IL within the nanoparticles, does not interfere with rutin release. In addition, since no relevant skin permeability was observed, and no toxicity was verified in the study of cell viability in HaCat, human keratinocytes, these nanocarriers may be suitable to be used for skin topical applications.

Additionally, all results obtained in this work seem to indicate that the IL-polymer nanoparticle hybrid system with [Cho][Phe], as choline-based IL, and with PLGA 50:50 is the best formulation for the delivery of rutin. The results also revealed the high potential of these new delivery systems to deliver poorly soluble drugs, since the incorporation of the ILs in the nanocarrier contributed positively to the stability of the polymer nanoparticles as well as allowed higher incorporation of the drug in the nanocarrier.

In conclusion, the produced IL-polymer nanoparticle hybrid systems may be used as a strategy to overcome the low solubility of some drugs, contributing to a higher drug loading and to a controlled and/or targeted delivery. The findings in this work, may open a new paradigm on the use of IL-polymer nanoparticle hybrid systems to deliver poorly soluble drugs, with clear benefits to the therapy of different diseases and health problems.

## Figures and Tables

**Table 1:** Freeze-drying ratio for the rutin-loaded IL-polymer nanoparticle hybrid systems, in the presence and in the absence of trehalose results, obtained from Equation (2). Data represented as mean ± SD (*n* = 3).

Polymer	IL	Freeze-Drying Ratio	
		No Lyoprotectant	Trehalose at 3% (*w*/*v*)
PLGA 50:50	[Cho][Phe]	1.31 ± 0.01	1.01 ± 0.01
	[Cho][Glu]	1.45 ± 0.05	1.04 ± 0.01
PLGA 75:25	[Cho][Phe]	1.28 ± 0.04	1.01 ± 0.02
	[Cho][Glu]	1.50 ± 0.04	0.98 ± 0.02

**Table 2:** Association Efficiency (AE) of rutin-loaded ILs-polymer nanoparticle hybrid system. Data represented as mean ± SD (*n* = 3). Results are significantly different (*p* < 0.05) between ILs for each polymer, when marked with *.

Polymer	IL	AE (%)
PLGA 50:50	[Cho][Phe]	75.6 ± 1.0 *
	[Cho][Glu]	53.8 ± 2.4
PLGA 75:25	[Cho][Phe]	73.2 ± 0.9 *
	[Cho][Glu]	51.3 ± 1.3

**Table 3:** Permeation flux of rutin IL solution, at 0.87 mg/mL for [Cho][Phe] and 0.37 mg/mL for [Cho][Glu], and rutin-loaded IL-polymer nanoparticle hybrid systems. Data represented as mean ± SD (*n* = 5).

Formulation	IL	Flux (µg/cm^2^/h)
Rutin solution	[Cho][Phe]	0.50 ± 0.09
	[Cho][Glu]	0.52 ± 0.08
PLGA 50:50	[Cho][Phe]	0.55 ± 0.13
	[Cho][Glu]	0.51 ± 0.11
PLGA 75:25	[Cho][Phe]	0.50 ± 0.06
	[Cho][Glu]	0.49 ± 0.12
